# Detecting the influence of rare stressors on rare species in Yosemite National Park using a novel stratified permutation test

**DOI:** 10.1038/srep10702

**Published:** 2015-06-02

**Authors:** J. R. Matchett, Philip B. Stark, Steven M. Ostoja, Roland A. Knapp, Heather C. McKenny, Matthew L. Brooks, William T. Langford, Lucas N. Joppa, Eric L. Berlow

**Affiliations:** 1 United States Geological Survey, Yosemite Field Station, 40298 Junction Drive, Suite A, Oakhurst, California 93644, USA; 2 University of California, Berkeley, Department of Statistics, Berkeley, California 94720-3860, USA; 3 United States Forest Service, Sierra National Forest, 1600 Tollhouse Road, Clovis, California 93611, USA; 4 University of California, Sierra Nevada Aquatic Research Laboratory, 1016 Mount Morrison Road, Mammoth Lakes, California 93546, USA; 5 National Park Service, PO Box 9, Denali National Park 99755, USA; 6 RMIT University, School of Mathematical and Geospatial Sciences, GPO Box 2476, Melbourne, VIC 3000, Australia; 7 Microsoft Research, Computational Science Laboratory, 21 Station Road, Cambridge, UK CB1 2FB; 8 Vibrant Data, Inc., 943 Clay St, San Francisco, California, 94108, USA.

## Abstract

Statistical models often use observational data to predict phenomena; however, interpreting model terms to understand their influence can be problematic. This issue poses a challenge in species conservation where setting priorities requires estimating influences of potential stressors using observational data. We present a novel approach for inferring influence of a rare stressor on a rare species by blending predictive models with nonparametric permutation tests. We illustrate the approach with two case studies involving rare amphibians in Yosemite National Park, USA. The endangered frog, *Rana sierrae*, is known to be negatively impacted by non-native fish, while the threatened toad, *Anaxyrus canorus*, is potentially affected by packstock. Both stressors and amphibians are rare, occurring in ~10% of potential habitat patches. We first predict amphibian occupancy with a statistical model that includes all predictors but the stressor to stratify potential habitat by predicted suitability. A stratified permutation test then evaluates the association between stressor and amphibian, all else equal. Our approach confirms the known negative relationship between fish and *R. sierrae*, but finds no evidence of a negative relationship between current packstock use and *A. canorus* breeding. Our statistical approach has potential broad application for deriving understanding (not just prediction) from observational data.

Drawing reliable inference from non-experimental data alone is notoriously difficult due to the potential for confounding influences^1^. This challenge is particularly acute in species conservation, where the goal is often to detect the influence of a human activity on a species’ distribution given the following constraints: a) The response variable of interest (e.g., an endangered species) is by definition rare; b) The potential stressor of interest may also be rare and not randomly distributed with respect to the species; c) Experimental manipulation to elucidate the relationship between potential stressor and species is either not permitted (e.g., due to low population size) or logistically impossible at the appropriate scale; d) Even a small negative response to the stimulus is of interest if there is any possibility to manage or control it; and e) Double rarity greatly increases the sample size needed to detect a relationship, when one exists. It is easier to use observational data for prediction than for understanding the relationship between a potential stressor and a response variable. For instance, generalised linear or additive models (GLMs or GAMs) fitted to observational data have successfully predicted a wide range of phenomena such as species occupancy on a landscape predicted from environmental data^2^,^3^, box office successes of movies predicted from Wikipedia activity^4^, and private personal traits predicted from Facebook “Likes”^5^. When using observational data for understanding rather than prediction, a standard approach to control for potential confounding factors is to fit a parametric model of the response based on all the predictor variables (including the stressor of interest), then to assess whether the coefficient of that stimulus in the fitted model is statistically significant. Disadvantages of this approach include: 1) It is important to limit model complexity by reducing the number of potential confounders by restricting the functional form of the model, or by other approaches to prevent over-fitting (e.g., insisting on smoothness, introducing a prior probability distribution for parameters, limiting the number and degree of interactions, etc.); 2) The form of the functional dependence of the response on the potential stressor needs to be specified; and 3) The stochastic model to justify significance testing is contrived and lacks any ecological interpretation. In other words, the terms in such a model may have nothing to do with the mechanism that generated the data, and the fitted model may be of little or no value in predicting the effect of a change in an individual predictor variable, that is, for causal inferences^1^.

We present a novel approach using observational data to infer whether a rare stressor affects a rare response variable by blending predictive models with nonparametric permutation tests. The predictive models, which use the best explanatory variables except the stressor of interest, are used to group cases into strata (based on predicted suitability for occupancy) that are comparable except for exposure to the stressor. There is no need to limit complexity of the predictive model or to specify its form. The goal is simply to do one’s best at predicting the response variable with available data without including the stressor (or covariates highly correlated with the stressor). A stratified nonparametric permutation test is then used to assess whether the stressor explains residual variation in the response variable and whether this observed association is, in aggregate, surprisingly large relative to that expected from the randomisation. We are not aware of any other method for causal inference from observational data that has fewer assumptions or ad hoc choices.

In the present work, we illustrate our approach with two case studies in Yosemite National Park (YNP), California, USA. The first case study, used to validate the approach, explores the known negative influence of non-native trout, introduced to naturally fishless lakes, on the occupancy of Sierra Nevada yellow-legged frogs (*Rana sierrae*) using a dataset of surveys conducted at 2,655 lakes (hereafter referred to as the “Lake” dataset). *R. sierrae* is endemic primarily to the Sierra Nevada mountains of California where it was once a common resident of lakes, ponds, and streams^6^. During the past century it has disappeared from more than 90% of its historical localities^7^ and, as a consequence of this precipitous decline, was listed as endangered under the United States Endangered Species Act (ESA) in 2014^8^. Although most of the native range of *R. sierrae* was naturally fishless, non-native trout have been widely introduced throughout its range to provide recreational fishing^9^,^10^. The strong negative influence of non-native trout on *R. sierrae* in lakes has been well documented, including in YNP, using both observational data^3^,^9^,^11^,^12^ and experiments^13^,^14^. Moreover, there is a well understood mechanism of influence (e.g., predation and competition). Both the stressor and response variable in this case are rare—each are present in ~10% of lakes (Fig. 1a.).

The second case study (and primary motivation for the current research) examines the influence of packstock use (typically horses and mules) in YNP meadows on the distribution of Yosemite toads (*Anaxyrus*[*=Bufo*] *canorus*) using a dataset of surveys conducted at 1,151 meadows (hereafter referred to as the “Meadow” dataset). Here we define occupancy by evidence of breeding. *A. canorus* was listed as threatened in 2014 under the ESA^8^, is endemic to the Sierra Nevada, and breeds in ephemeral, typically shallow, aquatic habitats associated with high elevation meadows^15^. Previous research suggests that *A. canorus* has disappeared from at least 50% of the sites where it occurred historically^16^,^17^,^18^,^19^. In the Sierra Nevada, packstock have been used for over 150 years to carry people and supplies^20^, and current recreational and administrative packstock use facilitates non-mechanised access to remote wilderness areas. On overnight trips, packstock are allowed to graze in meadows, some of which provide potential breeding habitat for *A. canorus*.

Unlike *R. sierrae*, the factors leading to the decline of *A. canorus* are poorly understood. Packstock grazing is one of many possible threats that have been proposed, along with airborne pesticides, infectious disease, climate change, habitat modifications, and livestock grazing^8^,^19^,^21^. There is a strong interest in understanding the influence of packstock on *A. canorus* because packstock use is controllable through management action, it could be ecologically significant for the survival of a rare species, and commercial packstock use in federally-protected wilderness has become a focal point of legal and political conflict^22^,^23^,^24^,^25^. Nearly all of YNP is federally designated wilderness. In a notable recent case, the High Sierra Hikers Association filed a lawsuit against the U.S. Forest Service (USFS) alleging that commercial packstock use in wilderness areas of the Inyo National Forest (adjacent to YNP) violated the National Forest Management Act, National Environmental Policy Act, and the Wilderness Act^23^. The court found in favour of the plaintiffs in part because the USFS had “failed to take a hard look” at potential negative impacts of packstock use on *A. canorus* before authorising special use permits for the commercial packstock operations^24^.

Proposed mechanisms for a potential negative impact of packstock on *A. canorus* include direct trampling of individuals and their aquatic breeding habitat, and indirect degradation of meadow hydrology^26^. In the latter case, disturbance of meadow vegetation by packstock^20^,^27^,^28^ is proposed to have similar effects as livestock (i.e., cattle and sheep), where grazing and trampling can result in the drying of meadows by causing erosion, incision of meadow stream channels, and lowering of the water table^29^,^30^,^31^,^32^,^33^. One recent experimental study examined potential local-scale effects of cattle grazing on *A. canorus* in a small number of meadows in the nearby Sierra National Forest and found no significant effect^34^,^35^, but it remains unknown whether packstock use has a real and important impact on *A. canorus* breeding occupancy across thousands of meadows throughout the species’ range.

Meadows are primary habitat both for toad breeding and for packstock grazing, but both are relatively rare. Similar to the case of frogs and non-native fish in lakes, ~10% of the meadows in YNP are occupied by toads or used for grazing (Fig. 1b.). For two reasons, this double rarity presents challenges for drawing reliable conclusions about the relationship between packstock and toads from occupancy data. First, packstock and *A. canorus* co-occur in only a small number of meadows. This pattern could suggest packstock have a negative effect on *A. canorus*; however, it could also be expected by chance because few meadows in the entire park have *A. canorus*. Second, *A. canorus* breeding is not randomly distributed across meadows because there are many factors other than packstock use—such as lack of breeding habitat, barriers to colonization, and deficiencies in any number of other resource requirements—that could determine the absence of *A. canorus* breeding.

To address these challenges, we used a previously-published predictive model^2^ of *A. canorus* breeding occupancy to first stratify meadows by predicted suitability for occupancy, and then a nonparametric permutation test to evaluate the question: *All else equal, does packstock use negatively affect A. canorus breeding occupancy in meadows across the entire Park?* We find that the chance of observing these patterns of recent packstock use and *A. canorus* breeding occupancy across thousands of YNP meadows would be extremely small if the relationship between packstock and *A. canorus* occupancy were in fact negative. This result was robust to several potential sources of confounding and uncertainty, which we discuss below.

## Results

Our stratified permutation method detected the known strong negative association between *R. sierrae* and non-native fish occupancy in lakes (Fig. 2). The observed difference in the proportions of fish-occupied lakes with *R. sierrae* and fishless lakes with *R. sierrae* was –0.0977 and was highly unlikely under the randomisation model (p-value = 0 — there were no cases in 10,000 permutations where the absolute value of the permuted test statistic was greater than the observed one).

In contrast to the *R. sierrae* versus fish occupancy patterns, the association between *A. canorus* breeding occupancy and packstock use in meadows was not different than that expected by chance under the randomisation model (Fig. 3). The observed correlation between maximum yearly stock nights and breeding was r = 0.051 (p = 0.080), and the correlation between average yearly stock nights per hectare and breeding was r = 0.045 (p = 0.115). When we took into account uncertainty in packstock use allocation to meadow polygons, we never observed a negative relationship, although for some configurations there was a weak positive correlation between toad breeding and packstock use that could be considered statistically significant (at a p ≤ 0.05 level) according to the randomisation model (Fig. 3c, f).

We explored whether the results for these two case studies were sensitive to various choices in the stratified permutation method including: a) using fewer strata, weighting or not the permutation probabilities within strata, b) weighting or not the strata in constructing the overall test statistic, and/or c) aggregating within-strata associations by averaging the raw versus absolute test statistic values (for example, negative associations in some strata might cancel out positive associations in others when using raw values, leading to no net association; whereas using absolute values would indicate an association, whether positive or negative, exists across the strata). All these alternatives yielded qualitatively identical results.

## Discussion

When both a potential stressor (here, non-native fishes and packstock) and the response of interest (here, *R. sierrae* and *A. canorus*, respectively) are sufficiently rare, there are not enough data to control for potential confounders by matching cases using cross-tabulation. We developed a new approach that matches cases, not on the raw values of the covariates, but on the predictions of an occupancy model based on those covariates. In other words, we collapsed multiple covariates known to potentially influence *R. sierrae* or *A. canorus* occupancy into a one-dimensional scale: the predicted suitability for occupancy. We used the predictive model—which does not include the potential stressor—to stratify cases on this scale, then applied a stratified permutation test to evaluate the association between the rare potential stressor and the rare response variable. This test assumes that, in the absence of the stressor, every case in a stratum is essentially equally likely to be occupied. If the strata are wide enough that there remains substantial variation among cases within strata, the probability of a case being assigned occupancy during a permutation can be weighted by the predicted suitability for occupancy.

Our method appears similar to propensity score matching^36^ (PSM), in that each tries to match cases by reducing a host of potential confounders to a single score that is used to match cases when evaluating whether a stressor (or treatment) matters. But PSM makes strong parametric, stochastic assumptions about how subjects are assigned to the stressor and fits a parametric model for the stressor assignment using all covariates but the response. In contrast, we make no assumption about how units are assigned to the stressor. We predict the response using all covariates but the stressor, then check nonparametrically whether, within groups that are relatively homogeneous with respect to predicted response, the association between stressor and response is *as if* at random. PSM is geared towards estimating the magnitude of the stressor effect, but requires strong assumptions to do so. Our method is geared towards testing whether the stressor matters at all, under less restrictive assumptions. In the case of the *A. canorus*, PSM would assume that whether a given meadow has packstock use is the outcome of a random trial in which the probability of having packstock use depends in a known way on that meadow’s covariates (e.g., size, elevation, etc.), where every meadow’s chance of having packstock use has the same functional dependence on the covariates. It then would fit a logistic model for that probability (ignoring which meadows are predicted to have toads), match cases based on the estimated chance that they have packstock use, and estimate the treatment effect (i.e., a difference in toad occupancy) from the matched cases. In contrast, our method builds a predictive model for which meadows have toads (ignoring whether they have packstock use), groups cases based on predicted toad occupancy, and assesses nonparametrically whether, within those groups, the connection between packstock use and toad occupancy is *as if* at random.

Our approach presented here has several advantages: 1) It can accommodate a predictive model of arbitrary complexity (there is no danger of over-fitting); 2) It does not require specifying the functional form of the predictive model nor of the dependence of the response on the potential stressor (or treatment); 3) It does not require a causal interpretation of the predictive model; 4) The randomisation has a more realistic ecological interpretation than traditional approaches because it draws from the observed distribution; 5) The method yields a rigorous nonparametric test, conditional on the stratification; and 6) The method appears to have the potential to perform well, even in situations where the stressor and response are both rare. In other words, this approach separates prediction of the response variable from understanding the contribution of the potential stressor, without making any assumptions about how it interacts with any other covariates.

Our approach successfully detected the known strong negative influence of non-native fishes on the occupancy of lakes by *R. sierrae* (Fig. 2). This influence has previously been well documented for *R. sierrae* in YNP^12^, as well as for the mountain yellow-legged frog species complex (i.e., *R. sierrae* and *Rana muscosa*) across the Sierra Nevada^3^,^9^,^11^,^13^. Fish eradication from lakes results in the recovery of frog populations^13^,^14^, and supports the negative association between frogs and fish obtained from landscape-scale observational studies. The known mechanism of interaction is direct predation of *R. sierrae* and *R. mucosa* by non-native fish and competition for prey^37^.

In contrast to the fish–frog patterns, our analysis suggests that the observed correlation of *A. canorus* breeding and packstock grazing among meadows would be extremely improbable (relative to the randomisation model) if the relationship between toad occupancy and current patterns of packstock use was in fact negative (Fig. 3). Our approach explicitly accounts for potential natural co-variation of packstock use and *A. canorus*. In other words, if people tend to bring packstock to meadows that also happen to be preferentially used by *A. canorus* for breeding, we would expect to observe a strong positive correlation between toad occupancy and packstock use. Thus, one might argue that observing no relationship (or a weak positive relationship) between packstock use and *A. canorus* indicates a negative influence of packstock. However, this issue is exactly what our stratified and weighted permutation approach is designed to address. By using the best available predictive model of toad occupancy of meadows in YNP and stratifying on model predictions, we explicitly control for existing co-variation in packstock use and *A. canorus* presence to explore whether, all else equal, there is a statistically significant association between current patterns of packstock use and *A. canorus* breeding occupancy.

We address below some possible sources of uncertainty in these conclusions and limits to the scope of inference due to errors in the data and assumptions of the statistical approach. We first address data-related issues in these conclusions that include sampling error, error in the stressor data, error in the response data, and uncertainty in the geographic location of the stressor.

*Sampling error*. For the Lake dataset, 100% of all mapped lentic habitat was surveyed. For the Meadow dataset, we sampled 82% of all mapped meadows with reported packstock use and 43% of all mapped meadows in YNP within the elevation range of packstock use. The vast majority of the un-surveyed meadows (91%) had low predicted suitability for occupancy^2^. The 1,151 meadows sampled spanned a wide range of geographic, hydrologic, and landscape attributes that are representative of the portion of the park that lies within the elevation range of packstock meadows.

*Error in the stressor data (e.g., non-native fish detection or packstock use reporting)*. For the Lake dataset, detection of non-native fish presence and absence (using gill nets in deeper lakes and visual surveys in shallow ponds) was 100% consistent among repeated surveys^9^. For the Meadow dataset, all commercial packstock use data are self-reported by pack outfitters, and there is inherent uncertainty in these data. There is also potential concern that there may be some bias of under-reporting. However, we have no evidence to suggest any systematic bias in this error with respect to packstock use levels. In other words, we believe that the likelihood of under-reporting is the same across both low and high levels of use. We conclude that it is unlikely that this known uncertainty would qualitatively change the patterns we observe because the relative rankings of meadows with respect to packstock use should remain relatively consistent despite uncertainty in the exact stock nights per meadows. Regardless, our results are conditional on the packstock data available.

*Error in the response data (e.g.,* R. sierrae *and* A. canorus *detection error*). For both *R. sierrae* and *A. canorus*, in lakes or meadows (respectively) where they were recorded as absent, there is some possibility that they were actually present but not detected on that survey. We have no evidence to suggest that this uncertainty varies systematically with non-native fish presence or packstock use. In other words, any known detection error for the response is likely the same across all levels of the potential stressor. Thus, we do not believe this error would qualitatively alter the patterns of association observed.

*Uncertainty in geographic location of the stressor (e.g., non-native fish assignment to mapped lake, or packstock use allocation to mapped meadow polygons)*. For the Lake dataset, the assignment of fish observations on the ground to lake polygons on the map were verified for each observation using GPS. However, as mentioned in the Methods, for the Meadow dataset there was known uncertainty in the allocation of reported packstock use to mapped meadow polygons because the former was derived from written records based on meadow names rather than GPS locations. There were eight named meadow complexes (groups of nearby meadow polygons that are all called the same name for reporting purposes) that were scored by YNP experts as being *highly uncertain* in the spatial allocation of reported packstock nights to specific meadow polygons. We explicitly explored whether this uncertainty could influence the observed relationship between toads and packstock by finding the packstock use allocations that produce the maximum and minimum correlation between toad presence and packstock use level. The uncertainty never yielded a significant negative association between toad breeding occupancy and current patterns of packstock use (Fig. 3).

We next address some uncertainties in our conclusions related to the statistical approach that include quality of the predictive model, covariation among the stressor and other predictors, and strata selection and cutoffs. In separate work, we explore the theoretical behavior of several variants of this test and its performance in simulations under various hypotheses.

*Quality of the predictive model*. The statistical approach invites one to *do your best* at predicting the response using all covariates except the stressor in order to match cases for the stratified permutation test. To match cases better, one might weight the within-strata permutations using the predictive model. We have not explicitly tested how the quality of the predictive model matters for successfully detecting a stressor effect when there is indeed an effect. If one uses a model that predicts poorly, the residual error of the response will be greater, so it may be more likely to find that the stressor has explanatory power. The test is designed to evaluate whether the residual error has a large association with the stressor. Both predictive models used in our analyses were of high quality, with accuracy rates (the sum of true positives and true negatives divided by the total sample size at the optimal cutoff point) of 85% for the *A. canorus* model and 91% for the *R. sierrae* model.

*Covariation of the stressor and other predictors*. The approach relies on good separation of the stressor and the covariates used in the predictive model. If the predictors include something equivalent to the stressor (either identical or re-scaled), the stressor cannot have any additional explanatory power. If the predictors, or combinations of the predictors, include something highly correlated with the stressor but not equivalent to it, there might or might not be any additional explanatory power. It is critical to use knowledge of the system (the science, not the math) to determine what is appropriate to include in the model. In our cases, none of the covariates were strongly correlated with either stressor (|r| < 0.35 and |r| < 0.17 for all associations between model predictors and non-native fish presence and packstock use, respectively). Similarly, neither stressor was predicted well using full models of all covariates (R^2^ = 0.31 and R^2^ = 0.10 for non-native fish and packstock use, respectively). Thus we feel confident that there was reasonable separation of stressor and other covariates in our case. Further theoretical explorations need to be done to understand the consequences of different levels of correlation between the stressor and other predictors.

A somewhat similar issue is potential confounding of different stressors. For example, is it packstock *per se* that may be influencing toads, or is it actually the associated activity of human campers? In our study system, while indeed packstock use brings people to the same meadows, the vast majority of human use in the park does not involve packstock. Thus many meadows with no stock use receive significant backpacking use. For meadows that receive visits by both humans and packstock, there are a number of reasons why we might expect, *a priori*, that the direct impact of packstock on toad breeding might be greater than hiker impacts. For example, packstock wander and forage directly within wet meadows, while humans typically avoid walking or camping in wet areas where toads breed. Similarly, wilderness camping guidelines require hikers to camp and bury human waste far away from standing water sources, while packstock use patterns and behavior within meadows are not restricted. Fish-occupied lakes may also be expected to incur greater human impacts if they are associated with more people fishing, which may confound interpreting an association between fish and frog occupancy. However, within YNP, lakes were originally stocked with fish park-wide, independent of visitor use. An estimated 62% of lakes contained non-native fish by the early 1950s^38^, though stocking was limited to just 15 high-use lakes beginning in 1972 and ceased completely in 1991^39^. The lakes dataset (based on surveys conducted from 2000–2002) we used contained 245 lakes with fish, and most are remote enough to receive very little human use. Moreover, previous experimental research has established that direct interactions (predation and competition for prey) are the primary causes of lower *R. sierrae* occupancy within lakes having fish.

*Strata selection and aggregation*. The test has a number of *ad hoc* choices, such as the number of strata, the boundaries of the strata, and the method for combining results across strata to construct the overall test statistic. For instance, one might base the test on the across-strata maximum of the unsigned correlation within strata, on a weighted sum of the signed or unsigned within-strata correlations, or on some other scalar summary of the within-strata correlations. Which method of aggregation is most appropriate will depend on the scientific question of interest. We chose a final test statistic that is the un-weighted average of within-strata values because a) we wanted to know if there is an association between stressor and response, overall, at the scale of the entire park; b) there was no reason to prioritise an association in ‘good’ versus ‘bad’ habitat since there is a strong interest in protecting *A. canorus* regardless of the predicted suitability for occupancy; and c) if we tested different strata separately, we would have to correct for multiplicity, which would reduce the power to detect a real effect. Aggregating across strata in this way might hide a real effect if different strata had strong associations, but of varying signs, so that the overall association would be weak or non-existent. We investigated whether there was evidence of a strong unsigned association within strata and found none.

These results do not imply that there is no possible way (use levels or meadow conditions) for packstock to negatively influence *A. canorus* occupancy. For example, direct mortality of *A. canorus* individuals from trampling by livestock has been observed^26^, and it is also possible that under the right combination of conditions—such as packstock use levels, seasonal timing of use, year of use, and meadow type—packstock could negatively impact *A. canorus* meadow occupancy. However, our results suggest that this potential has not manifested itself at the scale of current *A. canorus* occupancy patterns across the entire park under recent patterns of stock use in space and time. Since our predictive model is based on occupancy data since 1992, we cannot evaluate whether longer term, historic patterns of packstock use have influenced current *A. canorus* occupancy patterns. In other words, some meadows in the model training set may have lacked *A. canorus* due to the ‘ghost of grazing past.’ Thus the spatial and temporal scope of inference for our conclusion is for patterns of packstock use since 2004 and *A. canorus* occupancy since 2009 within YNP.

Several studies have reported that *A. canorus* is declining across its range. For example, *A. canorus* is now absent from most sites where it was found historically, both in YNP^19^ and on USFS lands surrounding the park^16^. In addition, precipitous declines in several *A. canorus* populations just outside of YNP were observed in the late 1970s^18^. The causes of these declines remain uncertain, although some were associated with an unknown disease^18^,^40^. However, packstock grazing was not a cause of the decline in *A. canorus* because the declines occurred in populations inhabiting meadows that were not grazed. Similar to our failure to detect a negative effect of packstock grazing on *A. canorus*, two recent, experimental studies failed to find any effect of current cattle grazing levels on short-term *A. canorus* occurrence or abundance^34^,^35^. In our study, we unfortunately do not have accurate estimates of *A. canorus* abundance at each meadow, and therefore are unable to make any conclusions regarding the relationship between packstock use and population abundance.

Our statistical approach could be more generally applied to problems where one seeks understanding from observational data (i.e., non-experimental) and the data violate key assumptions of traditional approaches such as parametric GLM or GAM modelling. Ecological data often violate these assumptions, and the spatial scale of many ecological questions is often too large for controlled experiments at the appropriate scale. Our approach is designed to test whether a stressor matters under weaker assumptions by leveraging the predictive power of these models without interpreting them causally. We still need to investigate how the statistical power of our methods compares to that of GLM and GAM when the assumptions of those methods hold, the conditions under which violations of the key GLM and GAM assumptions matter, and when our method gives results similar to those of GLM and GAM despite violation of key assumptions. For example, in the case of non-native fishes and *R. sierrae*, GAMs also detected the negative influence of fish on frog occurrence, perhaps because the influence is so large^3^,^12^. In general though, it is difficult to draw conclusions about any statistical test’s power without first making very strong assumptions about the nature of the effects. To evaluate the power of our approach, we would have to make the following assumptions: occupancy is random, independent across sites, and has a probability that depends on the stressor identically across sites (e.g., a one-unit increase in the stressor adds the same increment to occupancy, regardless of the values of all other covariates). Since these assumptions are unrealistic for our system, power estimates (e.g., based on simulations) that rely on such assumptions would not bring additional insight.

Structural equation modelling (SEM) is another approach that has been used in analyses of ecological datasets^41^ in an effort to overcome problems with causal inference and model parameter estimation/interpretation, particularly when causal relationships among variables are more complex (e.g., environmental variable *E1* causally influences environmental variable *E2*, and *E2* causally influences response variable *R1*; versus in a GLM where *E1* and *E2*, and perhaps their interaction, are modeled as directly affecting *R1*). While SEMs do indeed have the potential to clearly identify a relationship between a stressor and response within the context of other influencing variables, they suffer from many of the same limitations as GLMs and GAMs, especially in that they make strong assumptions about the functional forms of relationships between variables. Numerous *ad hoc* choices about the SEM’s structural form must therefore be made; however, prior experimental research elucidating the relationships and functional forms is often lacking, and variables for key paths in the structural model may not be available. Again, the approach we present here merely requires any occupancy model with reasonable prediction accuracy in order to assess whether the stressor of interest appears to matter.

In summary, we have presented a new nonparametric statistical method for exploring whether a variable *matters* in observational or experimental data, based on matching cases using predicted outcomes. The matching allows one to impose a weak *all else being equal but for the variable in question* condition in situations where randomisation and experimental intervention are impossible. We applied this approach to evaluate the influence of rare stressors on rare amphibians in YNP. The method correctly detects a known effect of fish on *R. sierrae.* In contrast, our results suggest that contemporary levels of packstock grazing in meadows of YNP do not have negative effects on the distribution of breeding by *A. canorus*. These results are consistent with the results of other recent studies that failed to detect any effects of cattle exclusion from entire meadows or portions thereof on *A. canorus* habitat, occupancy, or the density of early life stages. Therefore, causes of the past and ongoing decline of *A. canorus* breeding occupancy remain uncertain, but appear unlikely related to contemporary patterns of packstock use. Identifying these drivers of change will be critical to protecting this rare species.

## Methods

### General Stratified Permutation Approach

We used a novel, two-tiered, approach to explore the relationship between a rare potential stressor and the rare species. The approach is summarised here, with further details provided in each case study below. We first develop predictive occupancy models for the amphibian species of interest (*R. sierrae* and *A. canorus*) using all available data except the stressor. We stratified the lakes and meadows according to the model predictions of suitability for occupancy, then performed a stratified nonparametric permutation test for association between the stressor and the rare species: non-native fish and *R. sierrae* in lakes, and packstock use and *A. canorus* breeding in meadows. Stratifying using the predicted suitability for occupancy serves to control for a suite of factors other than fish/packstock that influence *R. sierrae/A. canorus* presence. The permutation test then answers whether, all things equal, the association between the stressor and the rare species is statistically surprising relative to the distribution of permuted associations. The null distribution of the test statistic was estimated by using 10,000 pseudo-random permutations of the occupancy labels within each stratum, and then averaging these stratum-level associations with equal weights to produce an overall test statistic across all strata for each permutation. The nominal p-value of the null hypothesis (i.e., the probability that the observed association between stressor and response is no different from that arising from arbitrary labelling of cases within each stratum) was the proportion of those permutations for which the absolute value of test statistic was greater than or equal to the absolute value of the observed statistic (i.e., a two-sided test). Calculations were performed using R version 3.0 software.

### ***R. sierrae*** and Fish

**Figure 1:**
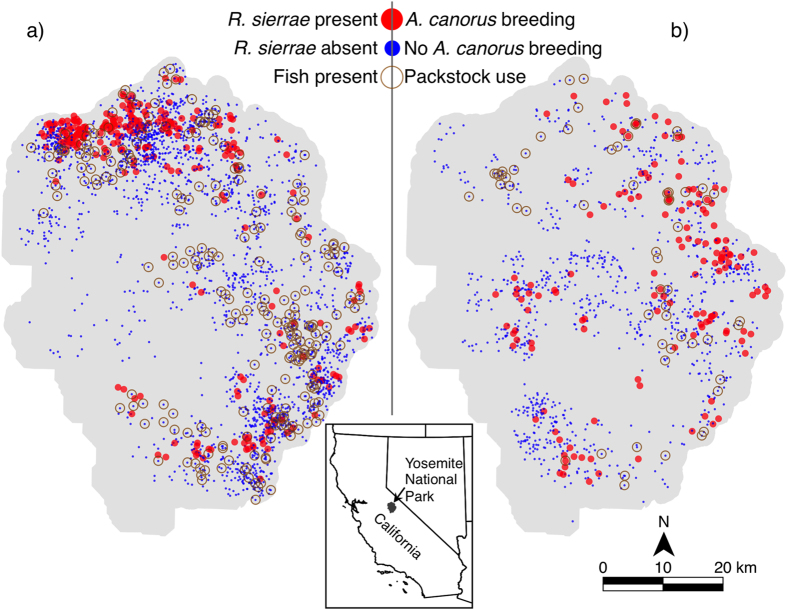
Map of Yosemite National Park showing the geographic distribution of two rare amphibians and their potential stressors across surveyed potential habitat patches in the park: (**a**) Sierra Nevada yellow-legged frog (*Rana sierrae*) and non-native fish occupancy in surveyed lakes, and (**b**) Yosemite toad (*Anaxyrus canorus*) breeding and packstock occupancy in surveyed meadows. The inset map shows the location of Yosemite National Park within California, USA. Random noise has been added to all point coordinates in order to obfuscate the precise occupied sites of these Federally protected species. Geospatial data were managed using PostGIS version 2.1 and maps composed using QGIS version 2.4.

**Figure 2:**
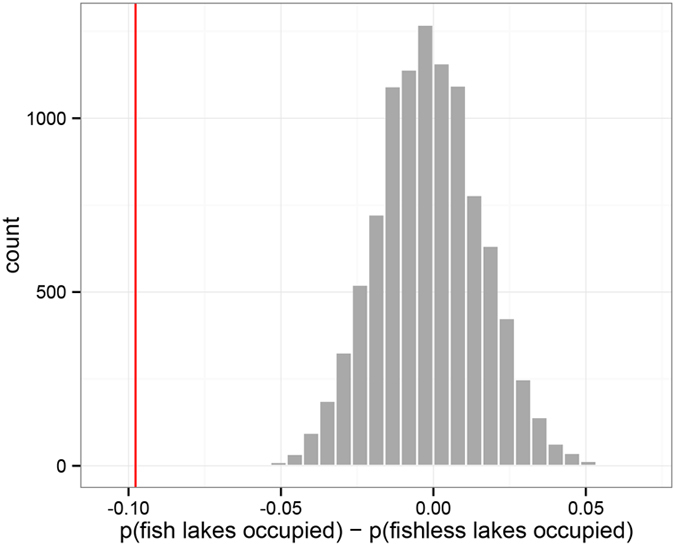
Results of the stratified permutation test examining the relationship between non-native fish and *Rana sierrae* occupancy in lakes of Yosemite National Park. The test statistic is the difference in sample mean occupancy between fish-containing and fishless lakes in the stratum (if fish negatively influence *R. sierrae*, we would expect this difference to be negative). Lakes were stratified by quintiles of predicted suitability of *R*. *sierrae* occupancy independent of fish. The red line is the observed test statistic, and the grey bars are the distribution of the test statistics from 10,000 permutations.

**Figure 3:**
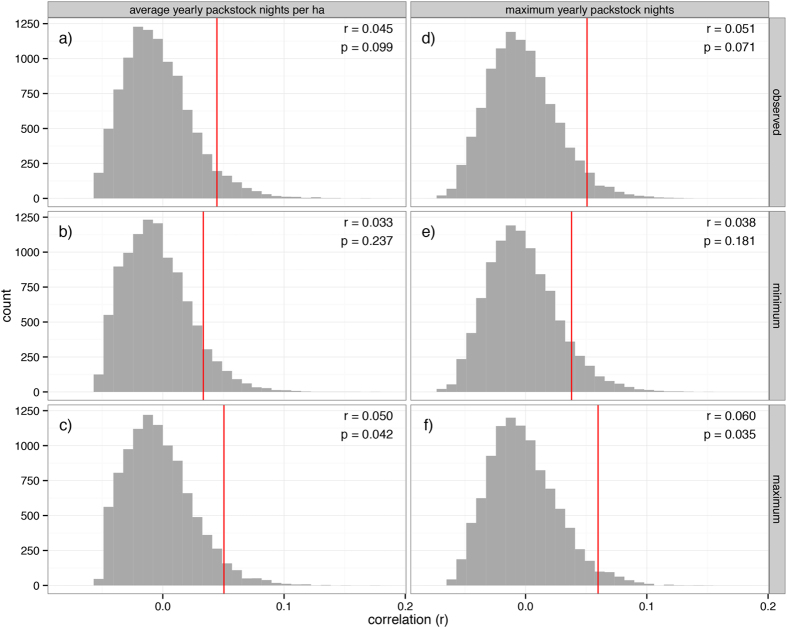
Results of the stratified permutation test examining the relationship between reported packstock use and *Anaxyrus canorus* breeding occupancy in meadows of Yosemite National Park for the two least correlated measures of stock use intensity: (**a**–**c**) average yearly packstock nights per hectare of meadow, and (**d**–**f**) maximum total yearly packstock nights. The test statistic is the point biserial correlation coefficient for the relationship between *A. canorus* occupancy and log packstock use. If packstock negatively influence *A. canorus* breeding, we would expect this correlation to be negative. The red line is the observed test statistic, and the grey bars are the distribution of the test statistics from 10,000 permutations. To evaluate the consequences of uncertainty in packstock use allocation to mapped meadow polygons, we present the minimum (**b** and **e**) and maximum (**c** and **f**) correlations observed from possible packstock use allocation combinations for meadow complexes with the highest uncertainty.

The Lake dataset consisted of surveys at 2,655 lakes conducted from 2000–2002 by Knapp^12^. *R. sierrae* (adults, juveniles, tadpoles, and/or egg masses) were present in 282 lakes, non-native fish present in 245, and *R. sierrae* and fish jointly present in 6 (Fig. 1a). We estimated suitability for occupancy of *R. sierrae* in lakes using the same predictive GAM developed by Knapp, with lake elevation, lake depth, shoreline substrate composition, dates surveyed, and lake spatial coordinates as predictor variables, but excluded the stressor (non-native fish occupancy) from the model. This occupancy model excluding the stressor had an overall prediction accuracy of 91%. We then stratified lakes according to the predicted suitability for occupancy of *R. sierrae*. Every lake in a stratum is thus considered to be roughly equally suitable to the rare species as any other lake in the stratum, except for the presence of the stressor, which is not explicitly in the model. Informally, if the stressor had no effect at all on the rare species, according to the occupancy model all the lakes in a stratum would be roughly *equally likely* to be occupied. If the stressor has no effect on the rare species, then the particular subset of lakes in the stratum that are occupied is no different than a random sample of that size from the lakes in that stratum. If the observed association between the stressor and occupancy within strata is surprisingly large compared to the association that would be observed if occupancy were assigned at random within each stratum, independently across strata, that is evidence that the stressor matters.

For fish and *R. sierrae*, we used five strata corresponding to quintiles of the predicted suitability for occupancy. We measured association by the difference in sample mean occupancy between fish-containing and fishless lakes in the stratum (if fish negatively impact *R. sierrae*, we would expect this difference to be negative). We then combined the differences in sample means across strata by taking their unweighted mean to produce the overall test statistic. If, overall, fish negatively impact *R. sierrae*, we expect the test statistic to be negative by an amount that would occur only rarely for random permutations of *R. sierrae* occupancy of lakes within strata, independently across strata.

### *A. canorus* and Packstock

The Meadow dataset consisted of packstock use records and *A. canorus* surveys throughout YNP. Packstock use data were compiled from records maintained by YNP. These records detailed the total number of stock nights (one stock night represents a horse or mule spending a night at a location) occurring within a meadow during a given year, and consisted of both administrative use (YNP staff) and commercial use (private companies authorised by YNP to conduct packstock trips). Stock use locations were reported using common meadow names, which we manually assigned to our mapped meadow polygon identifiers. In some instances a named meadow (e.g., “Tuolumne Meadows”) is a complex of several mapped meadow polygons, so we worked with YNP staff to allocate a meadow complex’s reported packstock use to the specific meadow polygons on the map. Confidence in these allocations varied, so we attributed each allocation with an ordinal level of certainty (very uncertain, moderately certain, or very certain), which we later used in our analyses to address the sensitivity of the tests to this uncertainty. Consistently recorded packstock use data were available from 2004 to 2012.

*A. canorus* surveys were compiled from those used in Berlow *et al.*^2^, plus surveys conducted from 2011–2012. We excluded meadows that were either outside the elevation range of reported packstock use or were surveyed prior to 2009 (the latter to ensure we had at least five years of reported packstock use data prior to each survey date). The resulting Meadow dataset consisted of 1,151 mapped meadows surveyed from 2009–2012, of which 935 were surveyed once, 191 were surveyed twice, and 25 were surveyed three or more times. Of these 1,151 meadows, 10 had both *A. canorus* breeding and packstock use, 137 had only *A. canorus* breeding, and 51 meadows had packstock use but no *A. canorus* (Fig. 1b). Our surveys covered 43% of all mapped meadows in YNP within the elevation range of packstock use and ~82% of all meadows with reported stock use that occur within the known elevation range of *A. canorus*.

Packstock use can be represented using several possible metrics, such as the maximum of annual total stock nights, the mean of annual total stock nights, or the cumulative sum of annual stock nights. Additionally, each of those can be divided by the meadow area to produce a measure of stock-use density. These metrics were all highly correlated (r = 0.61 to 1 for all pairwise comparisons) so we conducted separate analyses using two of the least correlated (r = 0.61) metrics: 1) maximum annual total stock nights and 2) average annual total stock nights per hectare. To consistently characterise the stock use level with respect to *A. canorus* occupancy for each sampled meadow, we summarised reported packstock use across the five years prior to and including the year when a meadow was surveyed for toad breeding. The surveyed packstock meadows covered a wide range of packstock use, with maximum annual total stock nights ranging from 0.700 to 295 (mean 44.5 and median 19.8) and average annual total stock nights per hectare ranging from 0.005 to 15.7 (mean 1.97 and median 1.02).

The distribution of packstock use values were highly skewed, with most meadows receiving no or light use and few meadows receiving very high use, so we used a log + 1 transformation of reported packstock use in our model. We used a binary representation of *A. canorus* occupancy (0 = no breeding, 1 = breeding), considering a meadow occupied if it had an observation of either eggs or tadpoles in at least one survey. To measure the degree of association between *A. canorus* and packstock use, we calculated a point-biserial correlation coefficient (equivalent to the Pearson correlation between a continuous variable and a dichotomous variable represented by 0s and 1s) between *A. canorus* occupancy and log-transformed packstock use.

To control for other factors that influence toad occupancy, correlations were calculated individually within strata defined using predicted suitability for occupancy from the species occupancy model, a model that predicted *A. canorus* detection (and non-detection) with ~85% accuracy using a suite of 15 environmental variables (see Table 1 in Berlow *et al.*^2^). Meadows were stratified into 4 levels using quartiles of predicted suitability for occupancy scores. There were not enough cases to stratify more finely without leaving at least one stratum with no stock nights or no *A. canorus* occupancy. Since the strata quartiles were relatively coarse, we controlled for remaining variation among meadows in occupancy suitability by generating permutations with unequal probability: the assignment of *A. canorus* to a meadow was proportional to that meadow’s predicted suitability for occupancy.

We explored the sensitivity of the correlation between *A. canorus* occupancy and packstock use to uncertainties in stock use allocations among meadows in multi-meadow complexes by searching for stock use allocations that resulted in the lowest and highest possible average correlations. There were eight meadow complexes containing a total of 24 meadows in which stock use allocation to meadow polygons was rated ‘very uncertain’. Stock use for those meadows was randomly allocated to meadows within a complex using 10% increments. For example, if there were four meadows in a complex, some possible allocations might be 10-20-40-30%, or 30-30-10-30%, or 100-0-0-0%, etc. We ran 100,000 iterations of random allocations of packstock use and retained the cases that led to the minimum and maximum correlations. Permutation tests were then conducted on those datasets to determine how rare the observed correlation would be in those extreme cases.

## Additional Information

**How to cite this article**: Matchett, J. R. *et al.* Detecting the influence of rare stressors on rare species in Yosemite National Park using a novel stratified permutation test. *Sci. Rep.* **5**, 10702; doi: 10.1038/srep10702 (2015).
